# Effects of *Fusarium solani* and *F. oxysporum* Infection on the Metabolism of Ginsenosides in American Ginseng Roots

**DOI:** 10.3390/molecules200610535

**Published:** 2015-06-08

**Authors:** Xiaolin Jiao, Xiaohong Lu, Amanda Juan Chen, Yi Luo, Jianjun J. Hao, Weiwei Gao

**Affiliations:** 1 Institute of Medicinal Plant Development, Chinese Academy of Medical Sciences and Peking Union Medical College, Beijing 100193, China; 2 School of Food and Agriculture, University of Maine, Orono, ME 04469, USA

**Keywords:** *Panax quinquefolius*, root pathogen, chemical response, biodegradation

## Abstract

American ginseng (*Panax quinquefolius* L.) is a highly valuable herb widely used for medicinal treatments. Its pharmacologically important compounds are the ginsenosides, which are secondary metabolites in American ginseng root. The concentrations of ginsenoside in roots can be changed by fungal infection, but it is unclear what specific root tissues are impacted and whether the change is systemic. In this study, American ginseng roots were inoculated with two fungal pathogens (*Fusarium solani* or *F. oxysporum*) and the levels of six ginsenosides (Rb_1_, Rb_2_, Rc, Rd, Re, and Rg_1_) were then measured in the phloem and xylem around the discolored lesions and adjacent healthy areas of the root. Results indicated that the growth of *Fusarium* spp. was strictly limited to phloem, and correspondingly the ginsenoside concentration was only altered in this infected phloem. The concentration of Rg_1_, Rd, and Rc significantly changed in phloem tissues where *F. solani* was inoculated, while only Rg_1_ and Rd changed significantly after *F. oxysporum* inoculation. However, no changes of any ginsenoside occurred in either xylem or phloem tissue adjacent to the inoculation point. In addition, when two *Fusarium* spp. were grown on ginsenoside-amended Czapek medium, the majority of ginsenosides were depleted. Therefore, pathogenic *Fusarium* spp. may reduce ginsenoside levels by consuming them.

## 1. Introduction

American ginseng (*Panax quinquefolius* L.) is mainly cultivated in the United States, Canada, and China [1]. It is a highly valuable perennial herb and has been widely used in Asia for hundreds of years as a medicinal and dietary supplement [1]. American ginseng produces a group of dammarane-type saponins known as ginsenosides. Some of the common ginsenosides such as Rb_1_, Rb_2_, Rc, Rd, Re, and Rg_1_ are active compounds responsible for the multiple pharmacological functions attributed to the plant [2,3], including anti-aging [4,5], anti-obesity [4], anti-hyperglycemic [4], and anti-infection activities [6,7], promotion for sperm motility and progression [8], and antimicrobial activities against bacteria [9].

Ginseng roots growing in the field are susceptible to several soil-borne diseases. These diseases are primarily caused by fungal pathogens, including several species of *Fusarium* [10,11,12], (causing root rot or rusty root), *Rhexocercosporidium panacis* (causing rusty root) [13], *Cylindrocarpon destructans* (causing root rot) [14,15], *Phytophthora cactorum* (causing Phytophthora root rot) [14,16], and *Pythium* species (causing damping-off) [15]. Among these pathogens, *Fusarium solani* and *F. oxysporum* are highly aggressive fungi causing American ginseng root rot in the Beijing ginseng producing region of China [10]. Typical early stage symptoms of this disease are reddish-brown to orange-brown discolored areas on the root surface. As the disease develops, symptoms include dry rot in both exterior and interior root tissues, and loss of fibrous roots. The infection of the root tissues may be associated with changes in the ginsenoside contents [17].

Saponins play an important role in the chemical defenses of plants against pathogen infections [18]. Some soil-borne pathogens are inhibited by ginsenosides [19,20], but some of these pathogens can alter the ginsenoside contents in return [21]. Gao *et al.* [17] have demonstrated that the quantity of ginsenoside Re was significantly reduced in the phloem, but increased in the xylem of American ginseng roots severely infected with root rot. A significant change has been reported in the level of ginsenoside Rb_1_ in infected roots five days post-inoculation [21]. However, the changes in the ginsenosides profile at stages of infection longer than 5 days are not fully understood, as a longer period of infection by pathogen is considered to alter the chemical composition and medicinal value of American ginseng roots to a greater extent [17].

A previous study [12] showed that the concentrations of ginsenoside Rg_1_, Rd, Rb_1_, Re, Rc, and Rb_2_ are reduced in infected epidermal and cortical tissues of American ginseng roots with rusty root symptoms. This study was conducted using ginseng roots collected from ginseng fields. As in the field a major pathogen is often accompanied with or followed by multiple other microorganisms, either pathogenic to ginseng roots or not [11,14], it is difficult to conclude that the ginsenoside reduction is due to any specific pathogen. This makes identifying the specific fungi that directly cause the ginsenoside alteration difficult, as other microorganisms can also change the concentrations of antimicrobial compounds in plants [22,23]. In addition, it is not exactly clear in which part of root tissue these compounds can be altered by fungal infection. Thus, laboratory assays using pure cultures of target organisms (pathogens) under a controlled environment are necessary.

We have previously found that Rb_1_ was rapidly induced within three to five days of inoculation by *Fusarium solani* and *F. oxysporum*, but the contents of Re or Rg_1_ were not significantly altered [21]. This suggests that Rb_1_, instead of Re or Rg_1_, is likely the active compound that inhibits conidial germination of *Fusarium* spp. [21]. The six ginsenosides Rb_1_, Rb_2_, Rc, Rd, Re, and Rg_1_ are similar in structure—they are all dammarane-type triterpenoid saponins, and have a common biosynthesis pathway—but whether their responses to pathogen infection are similar is unclear. Such a speculation is comparable to the case of other phytoanticipins/phytoalexins that share the same type of chemical structure but usually differ in function [24,25].

In China, American ginseng roots with visible necrosis are usually not discarded because of the high economic value of the uninfected tissue parts. Although the diseased tissues are eventually removed before consumption, it is not clear whether the ginsenoside content is affected in the rest tissues adjacent to the diseased lesions. Thus we were interested in how and where the contents of ginsenoside change upon *Fusarium* infection. The objectives of this study were to: (i) determine the effect of *Fusarium* on ginsenoside contents of American ginseng root during an extended period of infection; (ii) determine whether the alteration of ginsenoside is directly caused by metabolism of *Fusarium* spp.; and (iii) if *Fusarium* is the sole reason for ginsenoside alteration.

## 2. Results and Discussion

### 2.1. Molecular Detection of Fusarium spp. in American Ginseng Roots

Seven days after inoculation with *F. solani* (strain F19), cream sparse mycelia were present on the root surface, originating from the inoculation point. The center of disease lesions appeared reddish brown. In the case of *F. oxysporum* (strain C1), the entire infected root parts were covered with flourishing floccose white mycelia. In order to distinguish the late stage effects from the early stage ones, we defined the late stage as when visible disease symptoms and thick plaque were observed on ginseng roots after fungal infection. These phenotypes usually occurred seven days after inoculation, and were not clearly visible in the early stage of infection, as previously described [21].

**Figure 1:**
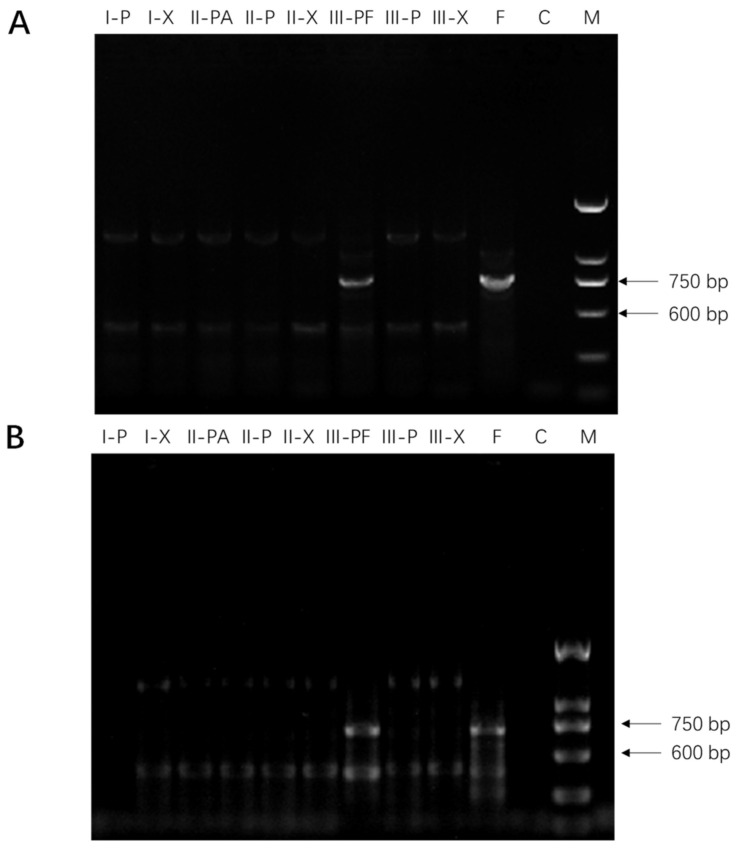
Detection of *F. solani* (strain F19, panel (**A**)) and *F. oxysporum* (strain C1, panel (**B**)) in American ginseng root tissues using polymerase chain reaction (PCR). Gel lanes show the presence of PCR products from left to right: (I-P), phloem of part I; (I-X), xylem of part I; (II-PA), phloem under PDA plugs of part II; (II-P), area adjacent to PDA plugs of part II; (II-X), xylem of part II; (III-PF), phloem under *Fusarium* culture plug of part III; (III-P), adjacent *Fusarium* culture plugs of part III; (III-X), xylem of part III; (F), positive control (*Fusarium* spp.), (C), negative control (no DNA template), and (M), DNA ladder.

The presence of fungi was only detected using polymerase chain reaction (PCR) in the phloem tissue directly under *Fusarium* mycelial plug (III-PF), suggesting that phloem cells of American ginseng root were infected upon contacting with fungal hyphae whereas the xylem cells were not affected. Interestingly, seven days after inoculation, the spread of fungi was restricted to the area with direct contact of fungal inoculums, not adjacent tissues in the phloem (III-P), despite the appearance of vigorous growth of fungal mycelia (Figure 1).

### 2.2. Alteration of Ginsenoside in American Ginseng Roots Inoculated with Fusarium spp.

Six ginsenosides, Rg_1_, Re, Rb_1_, Rc, Rb_2_, and Rd, were detected using high performance liquid chromatography (HPLC) and quantified by comparing them to standards. The corresponding HPLC calibration curves for Rg_1_, Re, Rb_1_, Rc, Rb_2_, and Rd were: *Y* = 216,944*X* + 5432 (0.0163–4.16 μg, *R*^2^ = 0.9996), *Y* = 198,382*X* + 52,512 (0.129–33.1 μg, *R*^2^ = 0.9993), *Y* = 177,784*X* + 52,102 (0.127–32.5 μg, *R*^2^ = 0.9995), *Y* = 173,823*X* + 48,412 (0.132–33.9 μg, *R*^2^ = 0.9996), *Y* = 180,668*X* + 3131 (0.0155–3.96 μg, *R*^2^ = 0.9996), *Y* = 210,351*X* + 7109 (0.0177–4.52 μg, *R*^2^ = 0.9995), respectively, where *Y* is the peak area in HPLC, and *X* is the concentration of the corresponding compound.

**Figure 2:**
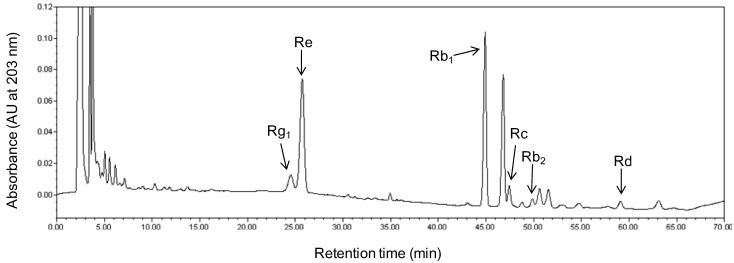
Representative retention peaks of a high performance liquid chromatography (HPLC) separation of ginsenoside Rg_1_, Re, Rb_1_, Rc, Rb_2_, and Rd, extracted from American ginseng roots.

**Figure 3:**
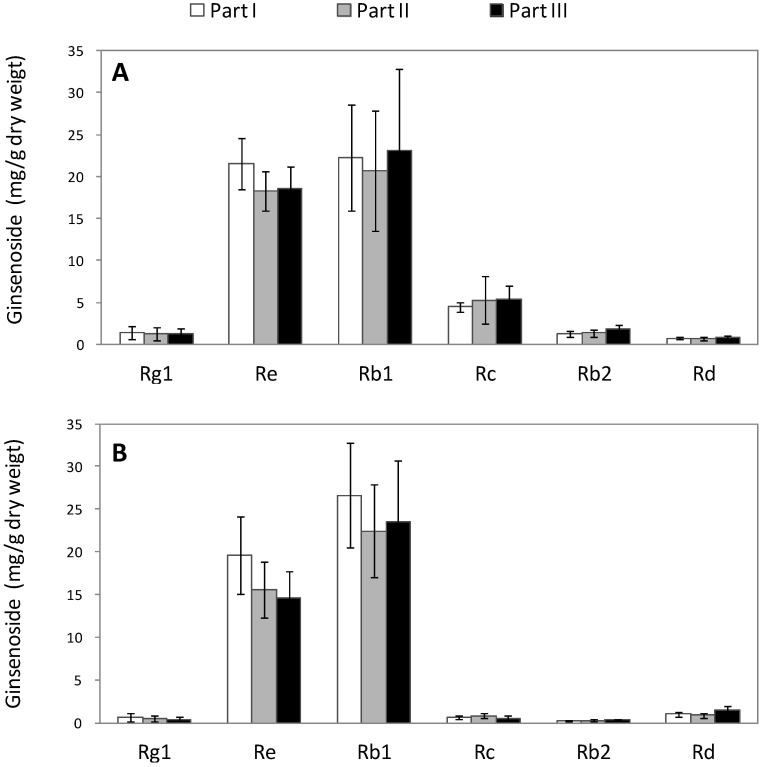
Measurement of ginsenoside (Rg_1_, Re, Rb_1_, Rc, Rb_2_, and Rd) contents (mg/g dry weight) in three root parts (I, II, and III) of healthy American ginseng in: (**A**) phloem and (**B**) xylem. Mean values of ginsenoside amongst the three root parts (I, II and III) are not significantly different at α = 0.05 (*p* > 0.05), analyzed using Fisher’s least significant difference (LSD) test.

The slopes of the six calibration curves followed the order Rg_1_ > Rd > Re > Rb_2_ > Rb_1_ > Rc, indicating that the HPLC detection sensitivities for the contents of these ginsenosides were reduced in this order. A representative HPLC elution profile of ginsenoside Rg_1_, Re, Rb_1_, Rc, Rb_2_, and Rd, in healthy ginseng roots is shown in Figure 2. The mean values ± SD of specific recovery rates with five replicates are (%): Rg_1_ 99.35 ± 1.18; Re 99.85 ± 1.76; Rb_1_ 97.25 ± 1.30; Rc 100.21 ± 0.43; Rb_2_ 97.89 ± 1.93; and Rd 95.11 ± 0.97. Regardless of the tissue type (xylem or phloem), the difference in ginsenoside content between the three ginseng root parts (part I, II, and III) was not significant in healthy ginseng roots (Figure 3).

No significant difference was found in contents of ginsenoside Rg_1_, Re, Rc, Rb_2_, and Rd among different root parts using two-way analysis (*p* > 0.05), while a significant difference was observed among different tissue types (*p* < 0.05) (Table 1). For the contents of Rb_1_, no significant difference was found either in root parts or in tissue types. Moreover, for all the six ginsenosides, no significant interaction was observed between root parts and tissue types.

**Table 1:** Summary of *p* values of the analysis of variance of ginsenoside contents in healthy ginseng roots.

	Rg_1_	Re	Rb_1_	Rc	Rb_2_	Rd
Part	0.698	0.051	0.659	0.656	0.071	0.079
Tissue type	0.001	0.011	0.406	0.000	0.000	0.005
Interaction between part and tissue type	0.998	0.898	0.831	0.676	0.235	0.398

**Figure 4:**
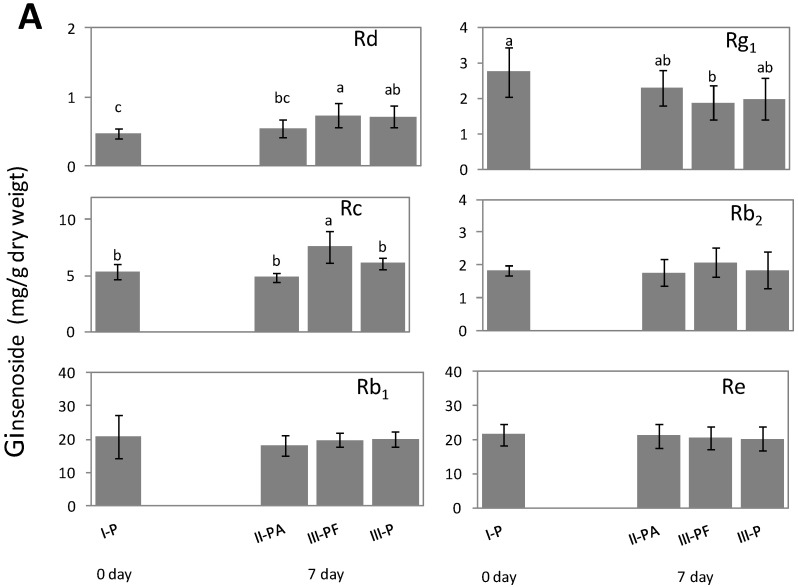

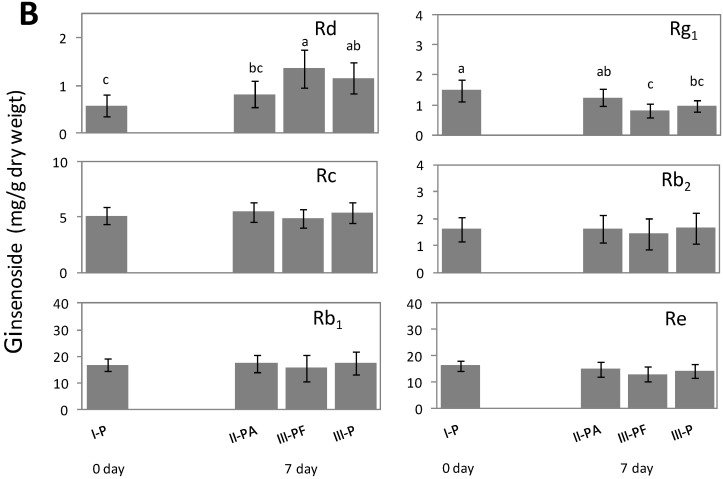
Contents of ginsenoside Rg_1_, Re, Rb_1_, Rc, Rb_2_, and Rd of American ginseng roots detected using HPLC in phloem tissues of the three parts (I, II, and III), including untreated (I-P), PDA-plugged (II-PA), *Fusarium* culture (III-PF) and untreated area adjacent to *Fusarium* culture (III-P). Samples were collected at 0 and 7 days after inoculation with (**A**) *Fusarium solani*; and (**B**) *F. oxysporum*. Mean values followed by the same letter or no letter were not significantly different (*p* < 0.05) in each ginsenoside (a panel in the figure), while followed by different letters were significantly different (*p* > 0.05), analyzed using Fisher’s least significant difference (LSD) test.

After seven days of treatment with PDA, the contents of all six ginsenosides in PDA plug-treated tissue (II-PA), including Rg_1_, Re, Rb_1_, Rc, Rb_2_ and Rd, were not significantly different from those in untreated tissue (I-P) at 0 days (Figure 4). After seven days of inoculation with *F. solani*, Rd and Rc in the infected phloem tissue (III-PF) significantly increased by 35% and 56% compared to that treated with PDA plug, respectively (Figure 4B). In the area of phloem tissue adjacent to the *Fusarium* inoculation spot (III-P), no lesions were observed, and the Rd and Rc contents remain unchanged.

*F. solani* infection significantly decreased the levels of Rg_1_ in the infected tissue by 18% compared to PDA plug treatment, while Rg_1_ did not change significantly in the area adjacent to *Fusarium* inoculation. However, infections by *F. solani* had no significantly effect on Re, Rb_1_, and Rb_2_ content in infected phloem tissues.

After seven days of inoculation with *F. oxysporum*, the content of the ginsenoside Rd in infected phloem tissue significantly increased by 65% compared to the PDA plug treatment (Figure 4B). There was also a trend of about 40% increase in the tissue adjacent to the *Fusarium* culture, but no significance was found compared to Rd content in PDA treatment. The content of Rg_1_ in the phloem tissues in direct contact with *Fusarium* culture was significantly decreased by 36% compared to PDA plug treatment. Rg_1_ content in the area adjacent to *Fusarium* inoculation also decreased by 23%, but no significant difference was detected when compared to Rg_1_ in PDA treatment. Moreover, infection by *F. oxysporum* had no effect on levels of Re, Rb_1_, Rc and Rb_2_ in infected phloem tissues.

**Figure 5:**
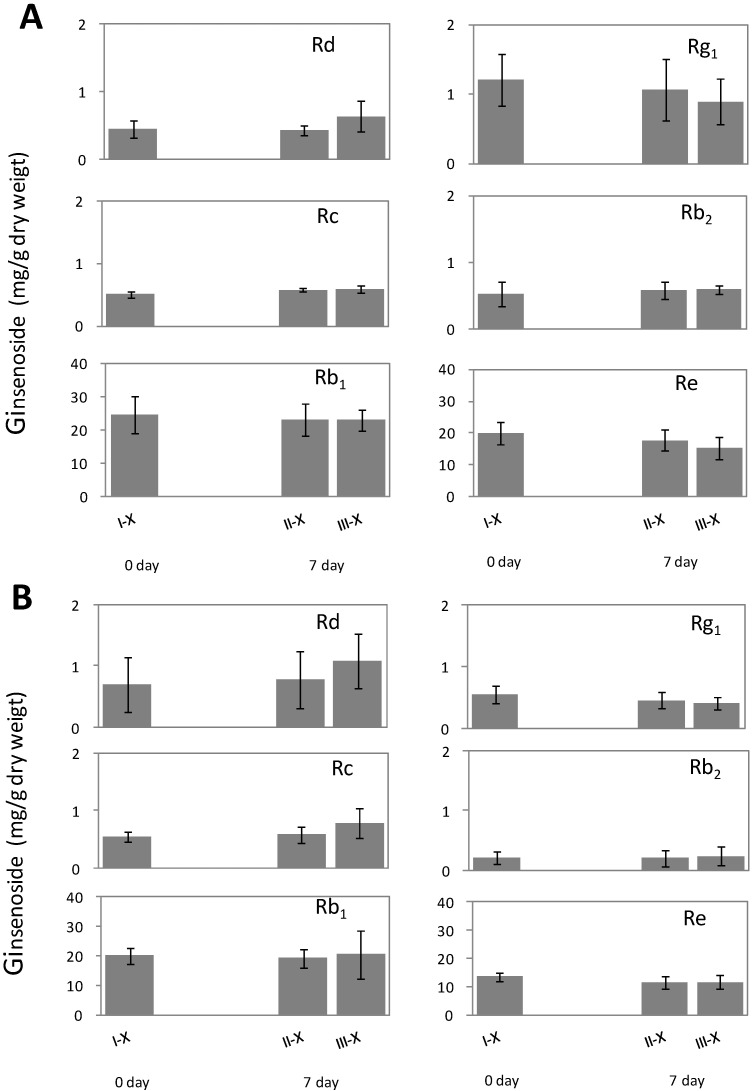
Measurements of ginsenoside Rg_1_, Re, Rb_1_, Rc, Rb_2_, and Rd in xylem tissue of American ginseng roots after inoculation for 0 or 7 days with: (**A**) *Fusarium solani* and (**B**) *F. oxysporum*. Root treatments include untreated (I-X), PDA plug (II-X), and *Fusarium* culture (III-X). Bars of mean values of ginsenoside with no letters indicate no significant difference (*p* > 0.05), analyzed using Fisher’s least significant difference (LSD) test.

Ginsenoside contents were also measured in the xylem of ginseng roots after inoculation of *F. solani* or *F. oxysporum*. Infection by either of the two *Fusarium* species did not affect the level of any of the six ginsenosides in the corresponding xylem tissues (Figure 5).

### 2.3. Metabolic Effect of Fusarium spp. on the Concentrations of Ginsenoside

After seven days of incubation on ginsenoside-amended Czapek medium, a white to cream coloration, and dense mycelia texture were observed in the group inoculated with *F. solani* or *F. oxysporum* conidia. All six ginsenosides were detected in the Czapek medium of *Fusarium* culture after seven days of incubation with *F. solani* or *F. oxysporum*.

**Figure 6:**
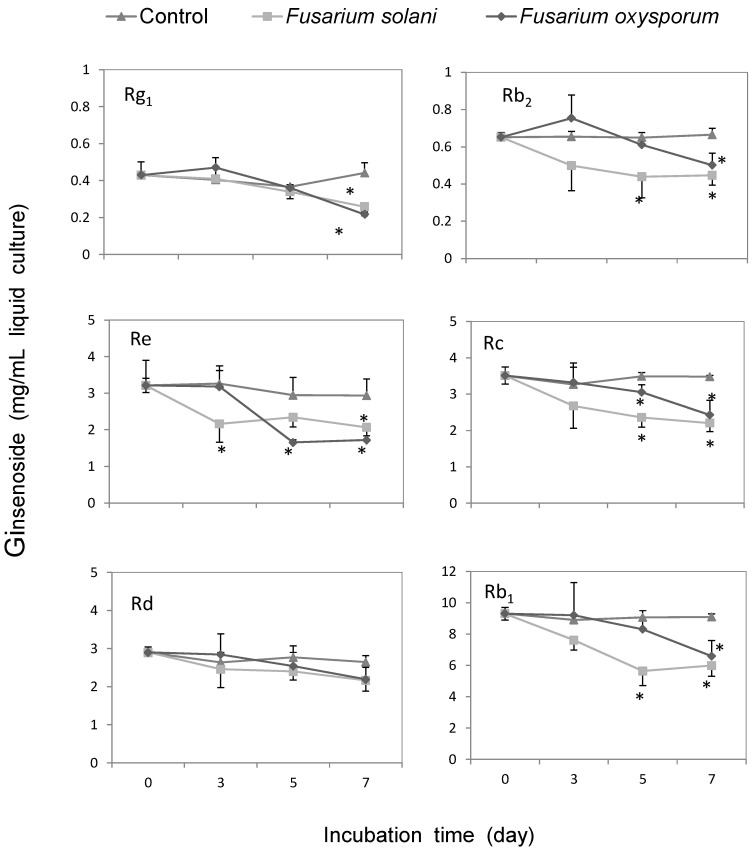
Metabolic effect of *Fusarium solani* and *F. oxysporum* on the concentrations of ginsenoside. Ginsenoside Rg_1_, Rb_2_, Re, Rc, Rd, and Rb_1_, were incubated with *F. solani* (square) or *F. oxysporum* (diamond) for 0 to 7 days. The values of ginsenoside content followed by an asterisk indicate a significant difference with control group (trilateral) at α = 0.05 (*p* < 0.05), as analyzed using Fisher’s least significant difference (LSD) test.

Compared to the amount initially added to the medium, Rg_1_, Re, Rb_1_, Rc, Rb_2_, but not Rd, were substantially depleted in the medium with *F. solani* or *F. oxysporum* (Figure 6). The degradation of these ginsenosides occurred after three to five days of co-culturing with the fungi (Figure 6). These results suggested that both *F. solani* and *F. oxysporum* were capable of inducing metabolic changes of major ginsenosides in American ginseng roots.

### 2.4. Discussion

We have found that *Fusarium* growth was strictly limited to the infected phloem tissues, as shown by the proliferation of *Fusarium equiseti* in American ginseng root [26]. After 7 days of inoculation, Rg_1_, Rd contents significantly changed in the phloem region tissues infected by both *F. solani* and *F. oxysporum*, but Rc content was only affected by *F. solani*, whereas Rb_1_, Re and Rb_2_ did not significantly change in *Fusarium*-infected phloem tissues. None of the ginsenosides was significantly altered in root phloem tissues where no *Fusarium* spp. mycelial growth was detected. This suggests that no direct chemical transduction occurs from infected phloem to xylem, and the ginsenoside alteration is likely a local reaction in American ginseng phloem tissue against *Fusarium* infection.

**Scheme 1:**
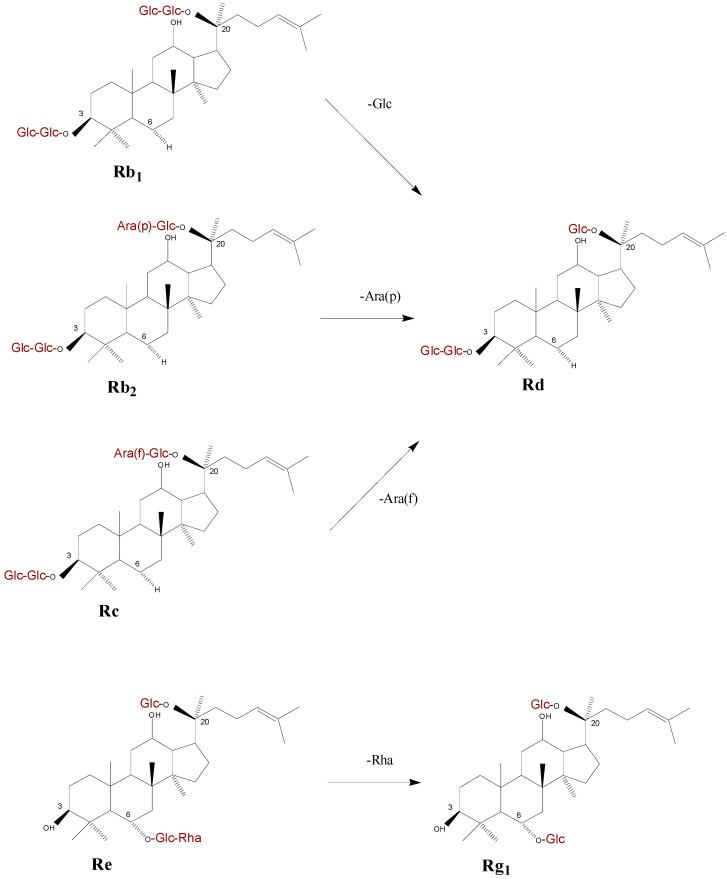
Chemical structures and transformations of ginsenoside commonly isolated from American ginseng roots according to Leung and Wong [3], and Yousef and Bernards [27].

Ginsenosides were biodegraded when co-incubated with *F. solani* and *F. oxysporum* cultures on Czapek medium, indicating a direct metabolism of ginsenoside by the fungi. This is in agreement with the fact that the contents of ginsenoside Rg_1_, Rd, Rb_1_, Re, Rc, and Rb_2_ were reduced by 9% to 44% in rusty root-affected epidermal and cortical tissues of American ginseng roots in an agricultural field [12]. In this study, the decrease of Rg_1_ in root tissues is likely attributed to the degradation by the *Fusarium* pathogens. Rc and Rd increased in American ginseng roots seven days after inoculation by the two *Fusarium* species. Rc increased in *F. solani*-infected American ginseng root tissues followed the up-regulation of ginsenoside Rb_1_ at 72 h after *F. solani* inoculation, showing a similar response with Rb_1_ to fungi infection [21]. Rd can be transformed from other ginsenosides, *i.e.*, Rc, Rb_1_ and Rb_2_, by removing terminal saccharide residues on C-20 (Scheme 1). Also, previous reports discovered that the terminal sugar units of ginsenoside Rc, Rb_1_ or Rb_2_ could be cleaved by glycosidase produced in many fungi, such as *Pythium irregulare* [27] and *Cylindrocarpon destructans* [25]. Moreover, pathogenic fungi such as tomato pathogen *Cladosporium fulvum* transformed ginsenoside Rb_1_ into Rd as a final product [28]. The *Fusarium* spp. used in our experiment might show the similar characteristic on ginsenoside decomposition. Here we suggest that the promotion of Rd in infected phloem tissues of ginseng root is probably derived from decomposition of other 20(*S*)-protopanaxadiol saponins such as Rb_1_ which was previously induced by the *Fusarium* spp. in early stage of infection [21]. Whether the increase of Rd in infected roots is correlated with its chemical defense against the two *Fusarium* spp. is still unclear. It will be of great interest for future studies to determine when and how these transformations occurred and are regulated under different pathogen infection status. *i.e.*, short term *versus* long term. We observed different responses in ginsenosides to the infection by *F. solani* and *F. oxysporum*: Rg_1_, Rc, and Rd were affected by *F. solani*, while only Rg_1_ and Rd were affected by *F. oxysporum*. Most likely the potentially different alteration of ginsenoside contents in ginseng roots is caused differently by the two *Fusarium* isolates’ infection. This type of difference has also been observed in *Leptographium wingfieldii* and *Ophiostoma canum* infected Scots pine resulting in different changes of monoterpene contents [29]. However, the specific mechanisms remain unclear and other *Fusarium* species still need to be examined.

The amounts of ginsenoside in infected American ginseng roots can be affected by both plant metabolism and fungal biodegradation, which may explain the different results seen in the ginsenoside alteration in ginseng roots and Czapek medium. Rc and Rd were increased in the *Fusarium-*infected root tissues, although they were biodegraded by the *Fusarium* spp. *in vitro*, showing a similar increased response as found for Rb_1_ in our previous study [21].

This likely indicates the synthesis of ginsenoside Rc and Rd were obviously induced by the two pathogen infections in the plant, and needs to be confirmed in further studies. Another reason for Rd increase is that it might be a metabolite of Rb_1_, Rc, and Rb_2_ formed by removal of terminal saccharide residues on C-20. Rb_1_ and Rb_2_ did not significantly change in the infected root tissues, so we hypothesize that the amount of synthesis of these compounds equals the amount degraded. However, this needs to be further confirmed. Rg_1_ significantly decreased in infected root tissues, while Re showed no significant change but rather a slight decrease. It has been confirmed that the syntheses of Rg_1_ and Re are not increased by the stimulation of *F. solani* and *F. oxysporum* [21]. Thus, once ginseng roots are infected, Rg_1_ and Re are degraded by the fungi. Due to the fact the content of Rg_1_ is lower than that of Re in ginseng root, the degraded amount caused a more notable effect on decrease of Rg_1_ than Re. Therefore, the content of ginsenoside in ginseng roots might be a combined consequence of pathogen metabolism and plant synthesis.

## 3. Experimental Section

### 3.1. American Ginseng Roots and Fungal Inoculums

Four-year-old healthy and fresh American ginseng roots were dug from a commercial farm in Beijing, China in October 2008. Roots of about 12 cm in length (equivalent to 15 to 20 g in dry weight) were carefully bagged, transported to the laboratory, and stored at 4 °C for later use. Cultures of *Fusarium solani* (isolate F19) and *F. oxysporum* (isolate C1) were obtained from reddish-brown discolored rotten areas of naturally-infected American ginseng roots in our previous study, and their isolation, identification, and pathogenicity were previously described [21]. The mycelium of each fungal isolate was transferred and cultured on potato dextrose agar (PDA: 200 g of potato, 20 g of dextrose, 13 g of agar, and brought up to 1 L with distilled water) plates at 25 °C for seven days before inoculation.

### 3.2. Root Treatments

Prior to the treatment, ginseng roots of approximately 12 cm in length were prepared by removing all the fine roots with a sterile scalpel. The taproots left were washed using tap water and then soaked in 75% ethanol for five minutes, followed by rinsing three times with sterile distilled water according to the previous report [21]. Each root piece was then transected into three sequential parts from the root tip to the base. The top part (part I), around three centimeters in length and eight grams in weight, was left untreated. The middle part (part II), around three centimeters in length and eight grams in weight, and the bottom part (part III), about five centimeters in length and also eight grams in weight, were disinfested by 3.25% NaClO for 10 min and then rinsed three times with sterile distilled water. Then part II was attached with pathogen-free PDA plugs on the surface as a control, while part III was inoculated by placing a mycelial plug (six millimeters in diameter) of each *Fusarium* isolate (F19 or C1) on the central surface of the root segment as a treatment. The treated root (part III) and corresponding root part II with PDA plugs were then incubated at 25 °C in the dark for seven days.

**Figure 7:**
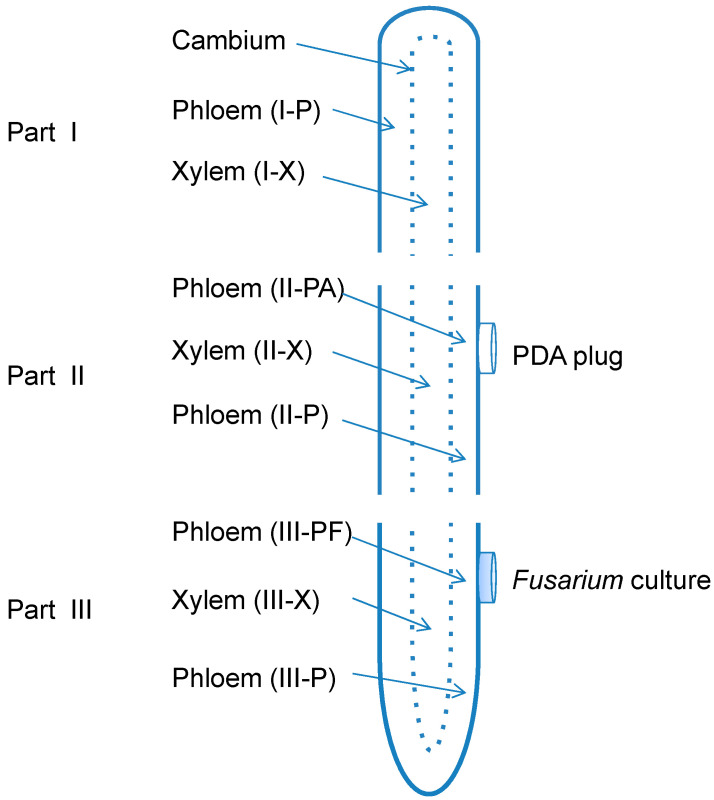
Schematic diagram of xylem (X) and phloem (P) tissues of a dissected taproot of American ginseng showing the three parts (I, II, and III) of sampling and inoculation with *Fusarium solani* or *F. oxysporum*.

For molecular diagnosis of the pathogen and analysis of ginsenoside contents, ginseng root parts I (the untreated group), II and III were peeled from the visible cambium. The outside tissue of cambium was considered as phloem, while the inside part was xylem. Moreover, phloem tissues underneath the mycelium of parts III (showing lesions) or control plugs of parts II (medium only) were separated from the adjacent phloem tissues. A total of eight different sample groups were analyzed, including phloem of root part I (I-P), xylem of part I (I-X), phloem under PDA plug of part II (II-PA) and adjacent area (II-P), xylem under the plug of part II (II-X), phloem under *Fusarium* culture plug of part III (III-PF) and adjacent area (III-P), and xylem of part III (III-X) (Figure 7). There were five replicates per treatment. All the eight groups were used for molecular detection of the two *Fusarium* spp. as described below, while seven groups were used for ginsenoside quantification, including I-P as background check, II-PA as a control, III-PF and III-P as inoculated groups of phloem; I-X, II-X, and III-X as background check, control and inoculated groups of xylem, respectively.

### 3.3. Molecular Detection of Fusarium spp. in American Ginseng Roots

To study the effect of fungal infection, we first confirmed that inoculation of American ginseng roots with both *F. solani* and *F. oxysporum* leads to consistent phenotypical changes. In order to investigate the ginsenoside production in different root tissues, the infected area in American ginseng root after inoculation by the two *Fusarium* species needed to be confirmed in this experiment. We used a PCR-based molecular detection assay to determine the extent of fungal infection in infected American ginseng roots. The treated root pieces were sterilized as described by Guo *et al.* [30], treated with 75% ethanol for one minute, then 3.25% NaClO for five minutes, followed by washing three times with sterile distilled water and once with 75% ethanol. The DNA was extracted using the CTAB method as previously described [31]. Briefly, root sections were crushed with a glass pestle in a mortar under liquid nitrogen, transferred into a 1.5 mL microcentrifuge tube containing 600 µL CTAB, and then incubated at 65 °C for 60 min. After centrifugation at 10,625 *g* for 10 min (1–14, Sigma Inc., Deisenhofen, Germany), the supernatant was transferred into a fresh 1.5 mL microcentrifuge tube, mixed with equal volume of Tris saturated phenol: chloroform: isoamyl alcohol at the volume ratio of 25:24:1, and centrifuged at 10,625 *g* for 10 min. The DNA in supernatant was then precipitated by isopropanol at −20 °C for 60 min, washed with 70% ethanol, and re-suspended into 20 µL sterile distilled water.

A part of the elongation factor 1alpha (EF*-*1α) gene was amplified with the primer pair EF1T 5′-ATGGGTAAGGAGGACAAGAC-3′ and EF2T 5′-GGAAGTACCAGTGATCATGTT-3′ [32]. Total DNA isolated from eight sample groups as described above together with the negative (no template DNA added) and positive controls (template DNA was isolated from mycelium of two *Fusarium* spp.) were amplified by PCR using the following settings of thermal cycler (Tprofessional, Biometra Inc., Göttingen, Germany): 94 °C for 85 s, followed by 35 cycles of 95 °C for 35 s, 57 °C for 55 s and 72 °C for 60 s, then a final extension at 72 °C for 10 min. Amplified DNA products were electrophoresed in 1% agarose gel in 1× TAE buffer (40 mM Tris-acetate, 1 mM EDTA, pH 8.0) at 100 V for 30 min. The gel was stained with Goldview (0.002% *v/v*, Tiangen Biotech Co., Ltd., Beijing, China), and examined under a UV light.

### 3.4. Quantification of Ginsenoside from Root Extracts

To better understand how ginsenosides are altered by *F. solani* and *F. oxysporum* at the late stage of infection, the contents of six major ginsenosides were analyzed in American ginseng roots using high performance liquid chromatography (HPLC, 600-486-717, Waters Inc., Milford, MA, USA) following the published method with slight modifications [33]. The root fragments from each treatment group were chopped into small pieces (approximately 1 × 1 × 0.5 cm) and dried at 45 °C for approximately three days, when consistent dry weight was obtained. Dried root pieces were then ground into powders using a blender (Linda Machinery Co., Ltd., Taizhou, China), sieved (sieve size opening 0.422 mm). An aliquot of 50 mL of water-saturated *n*-BuOH was added to 0.5 g of dry powdered roots to extract ginsenoside at 40 °C for 60 min with an ultrasonic extractor (Kunshan Ultrasonic Machinery Co., Ltd., Suzhou, China). The solution was filtered through a piece of quantitative filter paper (Special Paper Industry Co., Ltd., Hangzhou, China), and then 25 mL were transferred to an evaporating dish to dry. The dried evaporation residue was redissolved in 50% MeOH (aq) (5 mL) and filtered through a 0.45 μm nylon membrane filter (Jinteng Experiment Equipment Co., Ltd., Tianjin, China). The filtrated solution was stored at 4 °C for further analysis.

Standards of Rg_1_, Re, Rb_1_, Rc, Rb_2_, and Rd for HPLC were purchased from the National Institute for the Control of Pharmaceutical and Biological Products (Beijing, China). For HPLC elution, 10 μL of extracted liquid was injected into an Apollo^®^ RP C18 column (5 μm, 150 mm × 4.6 mm, Alltech Inc., Deerfield, IL, USA). For the analysis of five ginsenosides Rg_1_, Re, Rb_1_, Rb_2_, and Rd, the mobile phase was (A) acetonitrile and (B) 0.05% phosphoric acid (aq) with a flow rate of 1.1 mL/min as described below: 0 to 18 min, 21.5% A; 18 to 26 min, 21.5% to 28% A; 26 to 60 min, 28% to 34% A; 60 to 65 min, 34% to 21.5% A; and 65 to 70 min, 21.5% A [33]. With respect to ginsenoside Rc, the flow gradient of the same two mobile phases was slightly modified as: 0 to 25 min, 19% to 20% A; 25 to 41 min, 20% to 29% A; 41 to 46 min, 29% to 32% A; 46 to 71 min, 32% to 34% A; 71 to 73 min, 34% to 19% A; and 73 to 80 min, 19% A. The eluent was detected using an ultraviolet detector at 203 nm.

### 3.5. Metabolic Effect of Fusarium spp. on the Concentrations of Ginsenoside

To further investigate the direct effect of root pathogens on ginsenoside contents, *F. solani* or *F. oxysporum* mycelia were incubated *in vitro* with six ginsenosides (Rg_1_, Re, Rb_1_, Rc, Rb_2_, and Rd) on Czapek medium for up to 7 days and the contents of each ginsenoside in the culture medium were monitored using HPLC. To measure the effect of fungal infection on the stability of ginsenosides, fungal conidia of *F. oxysporum* and *F. solani* were incubated on ginsenoside-amended Czapek medium (2.0 g KNO_3_, 0.5 g KCl, 1.0 g K_2_HPO_3_, 0.5 g MgSO_4_∙7H_2_O, 30.0 g glucose in one liter distilled water). Specifically, 100 mg of crude ginsenosides (Hongjiu Lit. Co., Ltd., Dalian, China) dissolved in 0.3 mL methanol was mixed with 2.7 mL Czapek solution in a 6-well tissue culture plate (Corning Incorporated, Ithaca, NY, USA). Conidia of the two *Fusarium* species were obtained by harvesting six-day-old cultures on PDA in 10 mL sterile distilled water. The concentration of conidia suspension was adjusted to 1 × 10^6^ conidia/mL with a hemocytometer and 10 μL was added to ginsenoside-amended Czapek medium. Sterile distilled water was added in place of fungal suspension as a control. The culture plates were then incubated at 25 °C in the dark for seven days.

After seven days of incubation, mycelia were clipped out using tweezers, extensively wringed to release the culture solution, and washed in 0.5 mL distilled water three times. To recover the remaining ginsenosides, mixtures of spent culture solution and distilled water used in washing mycelia was extracted with water-saturated *n*-BuOH (3 mL) for five times. The combined *n*-BuOH phase was evaporated at 70 °C. The dry residue was dissolved in 5 mL 50% MeOH (aq) and filtered through a 0.45 μm nylon membrane filter. The filtrated solution was stored at 4 °C for further HPLC analysis by using five ginsenosides Rg_1_, Re, Rb_1_, Rb_2_, and Rd HPLC method as described above.

### 3.6. Data Analysis

Data were analyzed using statistical software SPSS (SPSS version 13.0; SPSS Inc., Chicago, IL, USA). Comparisons between the mean values of data of ginsenoside concentrations either in ginseng roots or in spent ginsenoside-amended Czapek medium were respectively performed using Fisher’s least significant difference (LSD) test, at the significance level α = 0.05. A two-way analysis was performed among the ginsenoside contents data in three parts (I, II, III) of two tissue types (xylem, phloem) of healthy ginseng roots. A p value < 0.05 indicates a significant difference.

## 4. Conclusions

We have found that both *Fusarium solani* and *F. oxysporum* affected the productions of ginsenosides in the infected roots of American ginseng, and ginsenoside changes occurred wherever *Fusarium* spp. reached or was present in the infected phloem tissue. Fungal biodegradation might be one of the reasons for the observed ginsenoside level alterations. The biology and biochemistry in uninfected root tissues did not change. It is unclear how saprophytic bacteria affect ginsenosides under natural conditions after root pathogen infection. This study provides a chemical basis for the response of American ginseng root to *Fusarium* pathogens, and also gives new insights for quality control of traditional Chinese medicinal plants.
