# Correction: Jiao et al. Effects of *Fusarium solani* and *F. oxysporum* Infection on the Metabolism of Ginsenosides in American Ginseng Roots. *Molecules* 2015, *20*, 10535–10552

**DOI:** 10.3390/molecules27123649

**Published:** 2022-06-07

**Authors:** Xiaolin Jiao, Xiaohong Lu, Yi Luo, Jianjun J. Hao, Weiwei Gao

**Affiliations:** 1 Institute of Medicinal Plant Development, Chinese Academy of Medical Sciences and Peking Union Medical College, Beijing 100193, China; jiao_1110@163.com (X.J.); luchauluchau@gmail.com (X.L.); impladroy@gmail.com (Y.L.); 2 School of Food and Agriculture, University of Maine, Orono, ME 04469, USA; jianjun.hao1@maine.edu

A reader recently brought to the attention of the Editor-in-Chief and the Editorial Office of *Molecules* several errors in our paper [1], with an allegation that they represented instances of deliberate image manipulation. In response to this complaint, we have now reviewed the original data, including the 2012 thesis of the co-author Jiao X. that was the basis for the reported results, as well as the additional data generated at the time for publication. We have also repeated some of the experiments following the described protocols, using both the original samples and, in some cases, new extracts obtained for this purpose. As a result of this review and new work, we wish to make the following corrections to the published article:

(1) Lane C without a loaded sample in Figure 1A,B in [1] was erroneously labeled as no-DNA negative control. To ensure the correctness of the reported results, instead of simply relabeling the old gel image, we have repeated the experiment and run a new gel. The mislabeled images should be replaced with the new gel images below. We believe that this error has no bearing on the conclusions of the paper.

(2) It was claimed that the HPLC ginsenoside quantification results in [1] did not match those in the thesis upon which the article was based and had therefore been manipulated. Rather than any intent to deceive, this is due to an error in our description of how the data were obtained. In [1], we erroneously stated that the quantitative analysis results of the six ginsenosides Rg_1_, Re, Rb_1_, Rb_2_, Rc and Rd in ginseng roots (Figure 3) were obtained using the HPLC gradient reported in Section 3.4 Quantification of Ginsenoside from Root Extracts, when in fact those conditions were used for the separation and quantification of the five ginsenosides Rg_1_, Re, Rb_1_, Rb_2_, and Rd, but not for ginsenoside Rc. As a result of a peer reviewer’s comment that the amount of Rc seemed unduly high, the original HPLC traces were re-examined in 2014 and it was noted that in several chromatograms the Rc peak was not resolved, resulting in erroneously high integration values in the thesis. The experiments were re-run using a modified solvent gradient that isolated the ginsenoside Rc peaks and gave lower values consistent with the literature and those results are the ones reported in [1]. Thus, for ginsenoside Rc, the sentence “The mobile phase was (A) acetonitrile and (B) 0.05% phosphoric acid (aq) with a flow rate of 1.1 mL/min as described below: 0 to 18 min, 21.5% A; 18 to 26 min, 21.5% to 28% A; 26 to 60 min, 28% to 34% A; 60 to 65 min, 34% to 21.5% A; and 65 to 70 min, 21.5% A [33]” should be replaced by “0 to 25 min, 19% to 20% A; 25 to 41 min, 20% to 29% A; 41 to 46 min, 29% to 32% A; 46 to 71 min, 32% to 34% A; 71 to 73 min, 34% to 19% A; and 73 to 80 min, 19% A”. We also believe that this error has no bearing on the conclusions of the paper. 

(3) All co-authors of the article have read and agree with the content of this Correction, except for Dr A. J. Chen, who in communications with the corresponding author and the Editorial Office has insisted for personal reasons on a retraction of the article rather than a correction. However, having examined the provided copies of HPLC traces and laboratory notebooks and the explanations provided by the authors, the Editor in Chief is satisfied that a correction without Dr Chen’s agreement and not a retraction is warranted for this paper.

The authors apologize for any inconvenience caused and state that the scientific conclusions are unaffected. The original publication has also been updated.

## Figures and Tables

**Figure 1:**
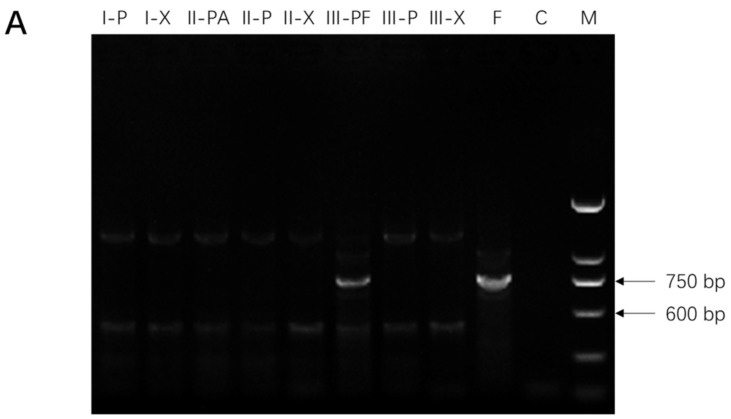

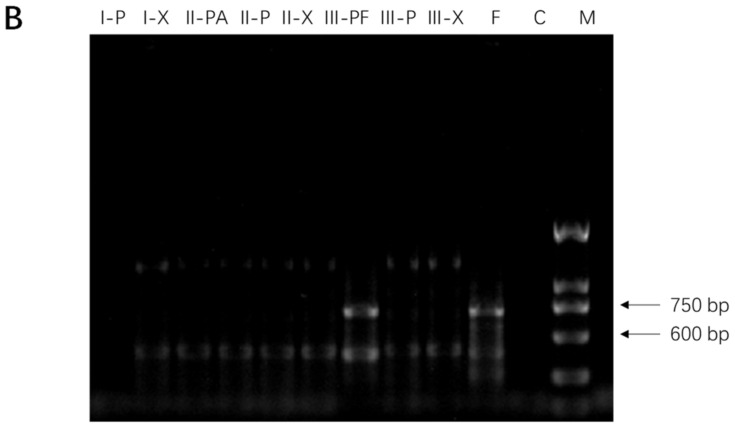
Detection of *F. solani* (strain F19, panel (**A**)) and *F. oxysporum* (strain C1, panel (**B**)) in American ginseng root tissues using polymerase chain reaction (PCR). Gel lanes show the presence of PCR products from left to right: (I-P), phloem of part I; (I-X), xylem of part I; (II-PA), phloem under PDA plugs of part II; (II-P), area adjacent to PDA plugs of part II; (II-X), xylem of part II; (III-PF), phloem under *Fusarium* culture plug of part III; (III-P), adjacent *Fusarium* culture plugs of part III; (III-X), xylem of part III; (F), positive control (*Fusarium* spp.), (C), negative control (no DNA template), and (M), DNA ladder.
